# 

**Published:** 1914-06-06

**Authors:** 


### Medical Appointments.

The following appointments have been made at the Manchester Royal Infirmary :—House physicians : Mr. W. R. Blore, M.B., Ch.B., Mr. G. W. Wood, M.B., Ch.B., and Mr. G. B. Jameson, M.R.C.S., L.R.C.P.; senior house surgeons : Mr. C. H. Crawshaw, B.A., M.B., Ch.B., M.R.C.S., L.R.C.P., and Mr. H. F. Hutchinson, M.B., Ch.B., M.R.C.S., L.R.C.P.; junior house surgeon : Mr. F. A. Bearn, M.B., Ch.B.; house surgeon to special departments : Mr. F. C. Bentz, M.B., Ch.B.; and medical registrar : Mr. T. H. Oliver, M.A., B.C., M.B., Ch.B.

The following gentlemen have been appointed physicians to out-patients at the West End Hospital :—Mr. Hugh Ridley Prentice, M.B., B.S. Lond., M.R.C.P., and Hildred Carlill, M.A., M.D. Cantab., M.R.C.P. Dr. Prentice was for some time resident medical officer at the National Hospital for the Paralysed and Epileptic. He received his training at St. Bartholomew's Hospital, was senior resident at the "Metropolitan," and is on the staff of the *Dreadnought* Hospital. He has translated Romer's book on "Epidemic Infantile Paralysis." Dr. Carl'll has been clinical assistant to the West End Hospital for Nervous Diseases, was a student at Guy's, medical registrar at "Westminster," and is also on the staff of the *Dreadnought* Hospital.

Rates of Subscription: Twelve months, 6/6; Six months, 4/-; Three months, 2/-, post free to any address in the United Kingdom. Abroad, Twelve months, 8/8; Six months, 5/-; Three months, 2/6, post free.—Address The Subscription Dept., "The Hospital," The Hospital Building, 28/29 Southampton Street Strand, London, W.C.

